# Mangiferin and oleocanthal in the modulation of oxidative stress in monocytes and macrophages

**DOI:** 10.1039/d6ra01563h

**Published:** 2026-07-08

**Authors:** Richa Sharma, Naga Venkata Anusha Anthikapalli, Renu Kushwaha, Alina Ovsii, Marek Rác, Pavel Pospíšil, Gabriella Tamasi, Agnese Magnani, Claudio Rossi, Ankush Prasad

**Affiliations:** a Department of Biophysics, Faculty of Science, Palacký University Šlechtitelů 27 Olomouc 779 00 Czech Republic ankush.prasad@upol.cz +420 585634752; b Department of Biotechnology, Chemistry and Pharmacy, University of Siena Via Aldo Moro 2 Siena 53100 Italy claudio.rossi@unisi.it

## Abstract

Oxidative stress and inflammation are tightly interconnected processes that contribute to the pathogenesis of numerous chronic diseases. In this study, we investigated the anti-inflammatory and antioxidant potential of two plant-derived bioactive compounds, mangiferin and oleocanthal, in human monocytic U-937 cells differentiated into macrophage-like cells. Cells were stimulated with phorbol 12-myristate 13-acetate (PMA), with or without lipopolysaccharide (LPS), to induce oxidative and inflammatory responses. The effects of mangiferin and oleocanthal (5 µM) were evaluated using cell viability assays, confocal microscopy, western blot analysis of tumor necrosis factor-α (TNF-α), 5-lipoxygenase (5-LOX) and electron paramagnetic resonance (EPR) spin-trapping spectroscopy for reactive oxygen species (ROS) detection. Both compounds were well tolerated and did not compromise membrane integrity or cell viability. Mangiferin and oleocanthal significantly reduced TNF-α expression and increased IL-4 expression in PMA and PMA/LPS-stimulated cells. While 5-LOX expression remained unchanged under PMA-induced differentiation alone, both compounds decreased 5-LOX levels under LPS-induced inflammatory conditions. EPR analysis confirmed suppression of ROS-derived radical formation following treatment with the bioactive compounds. Our results demonstrate that mangiferin and oleocanthal modulate oxidative stress and inflammatory signaling in macrophage-like cells by targeting TNF-α, 5-LOX, and ROS generation. These findings highlight their potential as natural therapeutic candidates for controlling inflammation-associated disorders.

## Abstract 2

Oxidative stress and inflammation are tightly interconnected processes that contribute to the pathogenesis of numerous chronic diseases.

## Graphical abstract

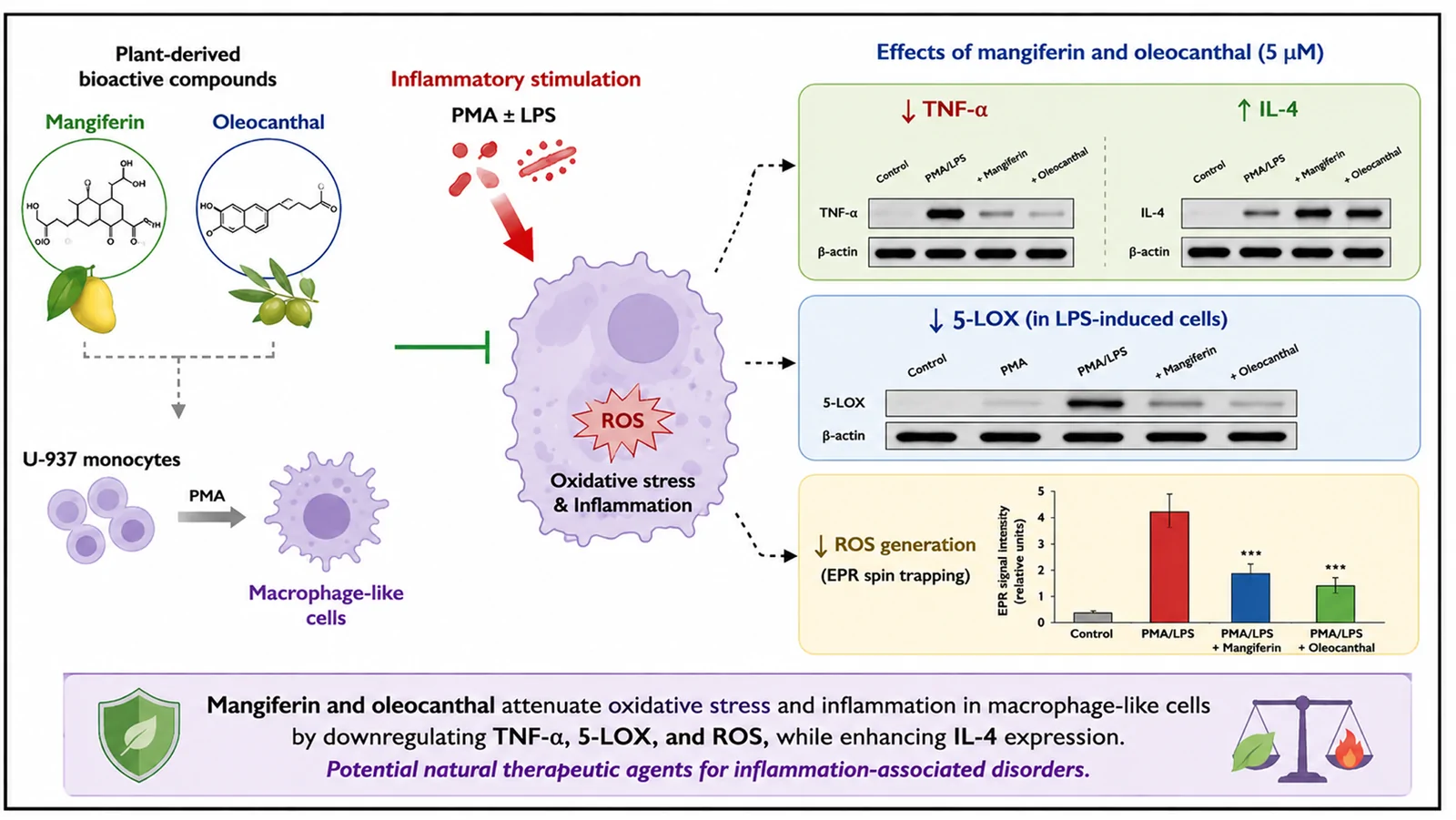

## 1. Introduction

Oxidative stress refers to a state of imbalance between the production of reactive oxygen species (ROS) and the ability of the antioxidant defense mechanism to neutralize them.^1,2^ Reactive oxygen species act as important signaling molecules that modulate cytokine production, inflammatory enzyme activity, and signaling cascades.^3,4^ Reactive oxygen species (ROS)-dependent mechanisms have been shown to influence both tumor necrosis factor-α (TNF-α) expression and 5-lipoxygenase (5-LOX) activation, two key mediators involved in macrophage-driven inflammatory responses examined in this study. TNF-α is a major pro-inflammatory cytokine produced by activated macrophages, whereas 5-LOX contributes to the generation of pro-inflammatory lipid mediators. Their redox-sensitive regulation further reinforces the close interconnection between oxidative balance and inflammatory signaling in macrophages.^2,5,6^ Since a typical diet may lack sufficient antioxidants to effectively combat intracellular ROS production, plant-derived bioactive compounds have gained increasing attention for their potential to modulate inflammatory pathways. These compounds can be sourced from agricultural by-products such as stems, leaves, husks, branches, seeds, and peels. As a result, bioactive compounds have become a major focus of research in recent years.^7–9^

Mango (*Mangifera indica* L.) and its associated by-products are rich sources of diverse phytochemicals with recognized biological activity. Among these, mangiferin, a prominent xanthone glucoside broadly distributed in mango leaves, peels, kernels, and other tissues, has attracted substantial scientific interest.^10^ Mangiferin has also been identified in coffee leaves. In particular, dry extracts obtained from Arabica coffee leaves have a Mangiferin content of approximately 4.4 mg g^−1^ dry weight.^11^ Mangiferin exhibits a wide spectrum of bioactivities, including antioxidants, anti-inflammatory, immunomodulatory and cytoprotective effects, which collectively position it as a promising natural therapeutic candidate. Mechanistically, mangiferin modulates several key molecular pathways involved in inflammation and redox regulation.^12^ It suppresses pro-inflammatory mediators such as TNF-α and cyclooxygenase-2 (COX-2) and influences transcriptional regulators including nuclear factor-kappa B (NF-κB) and nuclear factor erythroid 2-related factor 2 (Nrf2), thereby attenuating cellular inflammatory responses and enhancing endogenous antioxidant defenses.^13,14^ Its ability to reduce oxidative stress has been linked to the mitigation of metabolic dysfunction and protection against chronic disease associated tissue damage.^15^ Beyond its roles in inflammation and redox balance, mangiferin also interferes with cancer-related signaling, including the inhibition of caspase-3 activation, downregulation of matrix metalloproteinases (*e.g.*, MMP-7, MMP-9), and modulation of β-catenin pathways. In metabolic regulation, mangiferin activates AMP-activated protein kinase (AMPK), contributing to improved lipid handling, reduced adiposity, and enhanced glucose homeostasis.^16^ These properties support its potential relevance in conditions such as diabetes, obesity, and cardiovascular dysfunction. Despite broad pharmacological promise, the clinical translation of mangiferin is limited by its relatively low bioavailability. Ongoing research focuses not only on defining its mechanistic actions but also on improving its delivery and efficacy in biological systems.^17^

Oleocanthal is a phenolic secoiridoid abundant in extra virgin olive oil and is recognized as one of its most bioactive constituents. First identified as the compound responsible for the characteristic throat-pungency of high-quality olive oils, oleocanthal has gained attention for its broad spectrum of biological activities.^18^ Generated during olive processing through the enzymatic conversion of ligstroside- and oleuropein-derived precursors, oleocanthal displays notable bioactivity in inflammation, neurodegeneration, and cancer.^19^ A defining feature of oleocanthal is its strong anti-inflammatory potential, attributed largely to its inhibition of COX enzymes and suppression of downstream inflammatory mediators.^20^ In several *in vitro* systems, COX-inhibitory activity has been shown to exceed that of ibuprofen on an equimolar concentration, positioning oleocanthal as a naturally occurring compound with pharmacologically relevant anti-inflammatory actions.^12,21,22^ Beyond inflammation, oleocanthal exhibits antioxidant, neuroprotective, and chondroprotective properties, though clinical evidence remains limited.^18^ Oleocanthal has also emerged as a candidate of interest in neurodegenerative disease. Studies in microglial models demonstrate their ability to attenuate neuroinflammatory signaling, reduce oxidative stress, and enhance amyloid-β clearance, highlighting its potential relevance to Alzheimer's disease pathology.^22,23^ In oncology research, oleocanthal has demonstrated selective anticancer activity across multiple tumor types, acting through pathways that impair cancer cell survival, limit metastatic behaviors, and inhibit pro-angiogenic processes. Oleocanthal exhibits anti-melanoma activity and inhibits the STAT3 signaling pathway.^24^ Its anti-angiogenic actions include the blockage of endothelial cell proliferation, migration, and tube formation, mechanisms that may contribute to its tumor-suppressive effects. Together, these findings position oleocanthal as a key bioactive component of olive oil with diverse molecular targets and promising therapeutic potential, warranting further mechanistic and translational investigation.^22,25,26^

Different model systems have been used in the past to demonstrate efficacy and modulation by bioactive compounds, including mangiferin and oleocanthal. Immune cells exhibit distinct ROS profiles that reflect their specialized immunological functions.^27^ Phagocytic cells such as neutrophils and macrophages generate high levels of ROS through NADPH oxidase 2 (NOX2)-dependent oxidative bursts that support pathogen killing but can contribute to inflammatory tissue damage when overactivated. In contrast, lymphoid cells predominantly produce low level of mitochondria-derived ROS that function mainly in redox signaling and immune regulation. External stimuli further modulate ROS generation. Phorbol 12-myristate 13-acetate (PMA) induces a robust NOX2-mediated oxidative burst *via* protein kinase C activation, while lipopolysaccharide (LPS) enhances ROS production through toll-like receptor-4 (TLR4)-dependent inflammatory signaling.^28,29^

Human monocytic U-937 cells provide a widely used model for examining these processes because they exhibit strong, reproducible NOX2-driven ROS responses upon PMA or LPS stimulation and can be differentiated toward macrophage-like phenotypes.^30–32^ In our present study, U-937 cells were stimulated to induce an inflammatory and oxidative response, providing a controlled platform to evaluate the anti-inflammatory potential of mangiferin and oleocanthal, two bioactive natural compounds with reported modulation of redox and inflammatory pathways. Their effects were assessed using a combination of western blot analysis, to examine expression of key inflammatory mediators and complementary ROS measurements using electron paramagnetic resonance (EPR) spin-trapping spectroscopy, enabling a comprehensive characterization of their impact on cellular inflammatory signaling.

## 2. Materials and methods

### 2.1 Cell culture and *in vitro* differentiation

The human monocytic leukemia U-937 cell line was purchased from the American Type Culture Collection ATCC; Rockville, Maryland, USA). The cell lines were cultured in RPMI-1640 containing l-glutamine supplemented with 10% FBS and 1% (v/v) penicillin-streptomycin [Biosera (Nuaille, France)]. Cell lines were maintained at 37 °C in a saturated humidifying with 5% CO_2_. U-937 cells were seeded in 6-well plates at a density of 1 × 10^6^ cells per mL in a complete culture medium. To induce differentiation, the cells were treated with 250 nM and 150 nM PMA [Sigma-Aldrich (St. Louis, MO, USA)] for 72 hours in 6-well plate. PMA effectively drives U-937 cells to transition from their native monocytic state into macrophage-like cells. After differentiation, the cells were treated with the bioactive compounds (either mangiferin and oleocanthal) [Sigma-Aldrich GmbH (Mannheim, Germany], each administered at a final concentration of 5 µM, and incubated for an additional 24 hours. LPS (1 µg mL^−1^) [Sigma-Aldrich (St. Louis, MO, USA)^7^ was further added in cells treated with 150 nM PMA for 3 hours.

### 2.2 Cell viability assay

U-937 cell proliferation and viability were assessed using the trypan blue exclusion assay in control and differentiated cells, as well as after treatment with bioactive compounds in the absence or presence of LPS. In this method, trypan blue enters only cells with compromised membranes, staining non-viable cells dark blue, while viable cells exclude the dye and remain clear.^33^ To perform the assay, U-937 cell suspensions were mixed with 0.4% trypan blue at a 1 : 1 ratio and incubated for 2 minutes at room temperature. The stained samples were then transferred to a Bio-Rad dual chamber cell counting slide, and total, viable, and dead cells were quantified using a TC20 automated Bio-Rad cell counter.^7^

### 2.3 Confocal microscopy

The cellular morphology of the monocytic cell lines was visualized using an Andor BC43 Benchtop Confocal microscope (Oxford Instruments plc, Abingdon, UK). For all experimental samples, staining was performed using FM4-64 (Sigma-Aldrich GmbH, Mannheim, Germany), a water-soluble dye used to assess cell membrane integrity, and 4′,6-diamidino-2-phenylindole (DAPI) to visualize the nucleus. During sample preparation, a final of 15 µM for FM4-64 and 0.15 µg mL^−1^ for DAPI were used. Samples were incubated at room temperature for 5 min, then placed on glass slides and covered with coverslips prior to imaging. FM4-64 was excited using 561 nm laser and detected with 595/31 emission filter. DAPI, a nuclear counterstain that specifically binds to AT-rich regions in the minor groove of DNA, was excited using a 405 nm laser and detected with 442/20 emission filter. To validate the findings, SYTO9 staining was performed to evaluate cell membrane integrity and viability. Cells were incubated with SYTO9 + propidium iodide dye at a final concentration of 5 µM and 1.5 µM, respectively for 10 min at room temperature in the dark. Following incubation, fluorescence signals were detected using an excitation wavelength of 488 nm and an emission wavelength of 529/24 nm for SYTO9, and an excitation wavelength of 561 nm and an emission wavelength of 595/31 nm for PI.

### 2.4 Protein isolation

Cells were harvested by centrifugation at 1500 rpm for 10 minutes and simultaneously washed three times with 1× phosphate-buffered saline (PBS). Cell pellets were lysed in ice-cold Radioimmunoprecipitation Assay (RIPA) buffer [composed of 150 mM NaCl, 50 mM Tris–HCl (pH 8.0), 0.5% sodium deoxycholate, 0.1% SDS, and 1% NP-40, supplemented with 1% (v/v) protease and phosphatase inhibitors]. The cell suspensions were sonicated for 6 cycles with 40 amplitudes (30 s on ice between the cycles) followed by centrifugation at 14 000 rpm for 30 minutes at 4 °C to remove insoluble debris. The resulting supernatants, containing total protein extracts, were transferred to fresh microcentrifuge tubes and the total protein concentrations were quantified using the Pierce Bicinchoninic Acid (BCA) Protein Assay Kit (Thermo Fisher Scientific, Paisley, UK) according to the manufacturer's instructions.

### 2.5 Protein expression analysis by western blotting

Protein extracted from the cell pellets of each experimental group was analyzed by immunoblotting to assess the expression of β-actin, GAPDH, TNF-α, IL-4, 5-LOX and MDA. Antibodies were purchased from Proteintech (GmbH, Germany), and detailed information is provided in the SI Data 1. Except for 5-LOX, 10 µg of protein was mixed with 5× loading dye, heated at 70 °C for 10 minutes, and loaded onto a 10% Tricine SDS-PAGE gel. For 5-LOX, a total of 20 µg proteins were loaded. A pre-stained molecular weight marker (PL00001, Proteintech GmbH, Germany) was included to verify protein size. Following electrophoresis, proteins were transferred onto nitrocellulose membranes (Bio-Rad, California, USA) using the Trans-Blot Turbo transfer system. Membranes were rinsed with distilled water and blocked for 2 hours at room temperature with 5% skimmed milk in Tris-buffered saline with Tween-20 (TBS containing 0.1% Tween-20). The blocked membranes were incubated overnight at 4 °C with the appropriate primary antibodies. Excess primary antibody was removed by washing the membranes three times with TBST for 10 minutes each. Membranes were then incubated with HRP-conjugated secondary antibodies for 1 hour at room temperature, followed by another three washes with TBST. Signal detection was performed using the Immobilon Western Chemiluminescent HRP substrate (Sigma-Aldrich GmbH, Germany), and protein bands were visualized using the Amersham Imager 600 system. Band intensities were quantified using ImageJ software, and densitometric profiles are presented for statistical analysis.

### 2.6 Electron paramagnetic resonance (EPR) spin-trapping spectroscopy

EPR spectra of the POBN (4-pyridyl-1-oxide-*N-tert*-butylnitrone)-OH adduct containing 170 mM ethanol were recorded in induced U-937 cells, both in the absence and presence of bioactive compounds. Hydroxyl radicals were detected using 25 mM POBN *via* spin-trapping in a glass capillary tube (Blaubrand intraMARK, Brand, Germany). The cells were subjected to ultrasonic bath sonication during the final 30 minutes of incubation with the spin trap and/or LPS. Spectra were acquired on a MiniScope MS400 spectrometer (Magnettech GmbH, Berlin, Germany) under the following conditions: 10 mW microwave power, 1 G modulation amplitude, 100 kHz modulation frequency, 100 G sweep width, 1.62 G s^−1^ scan rate, and gain of 100.

## 3 Results

### 3.1 Cell proliferation and effect of bioactive compounds

**Fig. 1:**
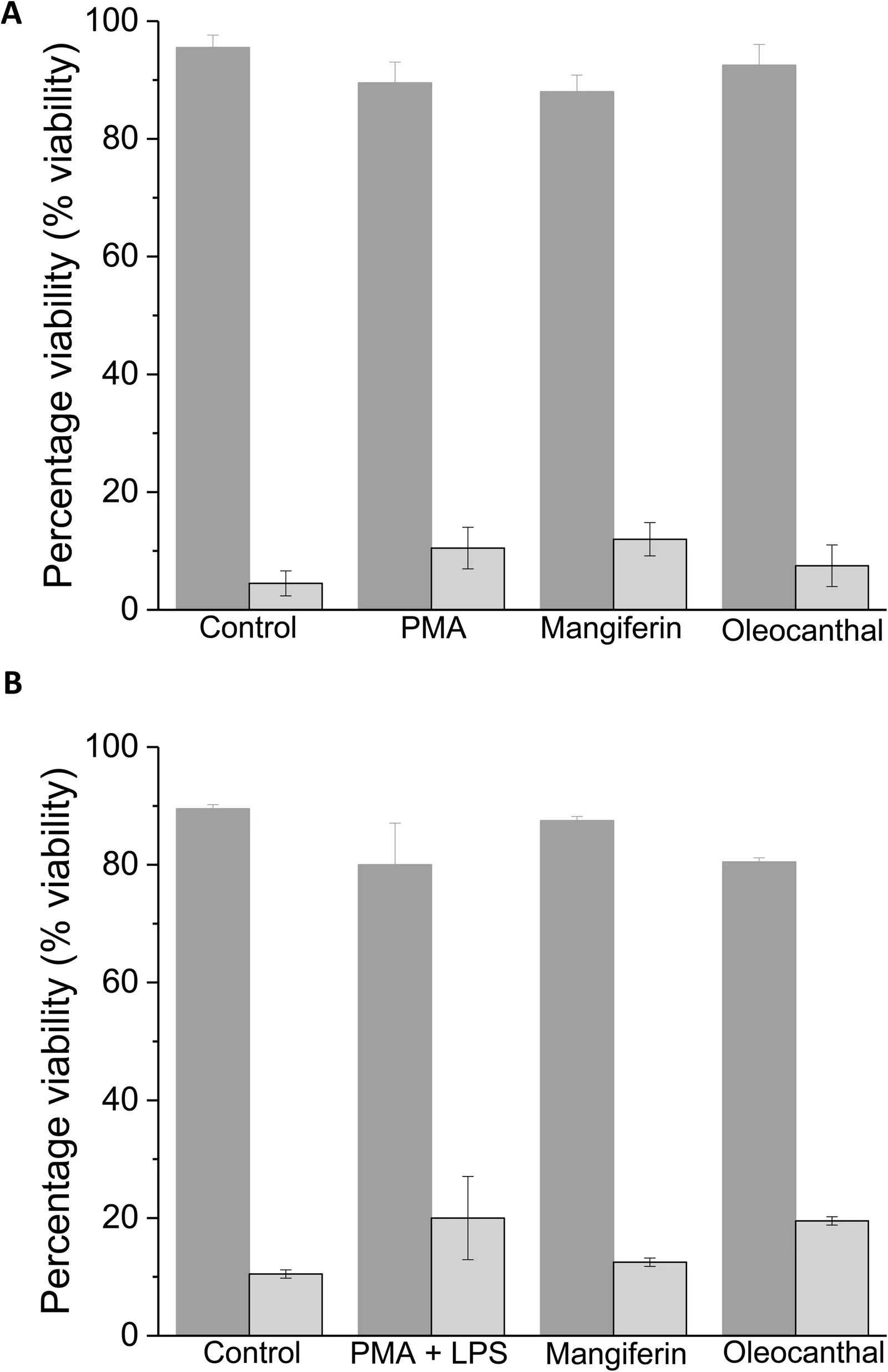
Cell differentiation was induced in U-937 cells using PMA (A) or PMA + LPS (B) at final concentrations of 250 nM (A) and 150 nM (B) PMA for 72 h, followed by incubation with bioactive compounds for 24 h. LPS was added during the final 3 hours. Cell viability was assessed using the trypan blue exclusion method. Bar graphs depict the live and non-viable cells, as determined by a Bio-Rad automated cell counter. In panels A and B, the bar graphs represent the percentage of viable (dark grey) and non-viable (light grey) cells. Values are presented as mean ± standard deviation.

Cell viability following PMA or combined PMA+LPS stimulation, in the presence or absence of bioactive compounds, was assessed using the trypan blue exclusion assay. The aim was to determine whether these treatments exerted any cytotoxic effects. Cells treated with PMA alone maintained high viability, with more than 80% viable cells observed across all experimental conditions (Fig. 1A). Similarly, co-treatment with PMA and LPS did not adversely affect cell viability, with viability consistently around 80% in all groups (Fig. 1B). Treatment with bioactive compounds also resulted in comparable viability levels. All experiments were performed in replicates, and statistical analysis revealed no significant differences in cell viability between treated and control groups.

### 3.2 Assessment of membrane integrity following PMA and LPS stimulation

**Fig. 2:**
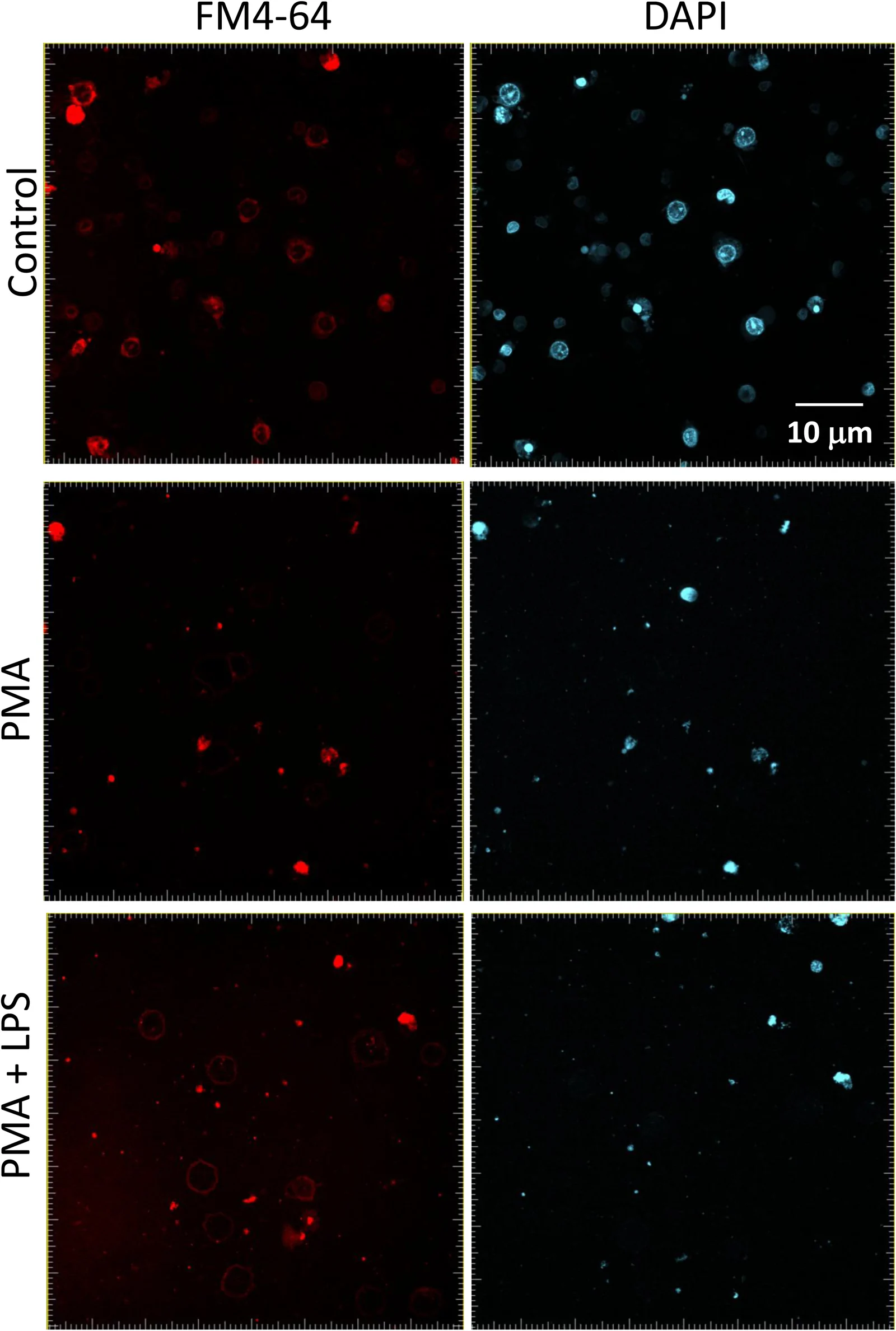
U-937 cells were double-stained with FM4-64 and DAPI following treatment with PMA for 72 h, followed by incubation with bioactive compounds for an additional 24 h and LPS treatment as described above. Multiple images were captured, and representative images (arranged from top to bottom) are presented for each experimental group: negative control (-PMA) and test groups (+PMA; +PMA and LPS).

In agreement with the trypan blue viability results, fluorescence staining using FM4-64 and DAPI further confirmed cellular integrity under all experimental conditions. FM4-64 membrane staining demonstrated continuous and well-defined plasma membranes, indicating preserved membrane integrity in cells treated with PMA alone and PMA+LPS. Concurrently, DAPI nuclear staining revealed intact nuclei with normal morphology, without evidence of nuclear fragmentation or condensation. These observations were consistent across all treatment groups, supporting the conclusion that the applied stimuli did not induce detectable structural or nuclear damage (Fig. 2).

To validate the findings, all non-treated and bioactive compound-treated U-937 cells were stained with two different dyes, SYTO9 and propidium iodide, to assess cell viability and monitor cell membrane integrity. SYTO9 is a green fluorescent nucleic acid stain that penetrates intact cell membranes and binds to DNA/RNA. Conversely, PI is a nucleic acid-binding stain that is membrane-impermeable and cannot diffuse through intact cell membranes of healthy cells. PI enters cells only through compromised cell membranes. Thus, yellow fluorescence represents non-viable or late apoptotic cells, indicating loss of membrane integrity.

Microscopic observation of all samples revealed that a substantial proportion of cells exhibited SYTO9 staining, indicating that most of the cell population in all samples was healthy and functionally active. Concurrently, a small proportion of cells showed PI staining, suggesting limited cell non-viability within the population. The low fraction of PI-positive cells indicates that PMA, LPS, mangiferin, and oleocanthal treatments did not trigger significant cytotoxic responses. The presence of some PI-positive cells may be attributed to baseline cell death and mechanical stress associated with sample handling. Overall, U-937 cells stained with SYTO9 and PI demonstrated high cell viability with only slight membrane compromise. The predominance of SYTO9-positive cells over PI-positive cells suggests that most U-937 cells-maintained membrane integrity under all experimental conditions (SI data 2).

### 3.3 Housekeeping genes and cell differentiation

**Fig. 3:**
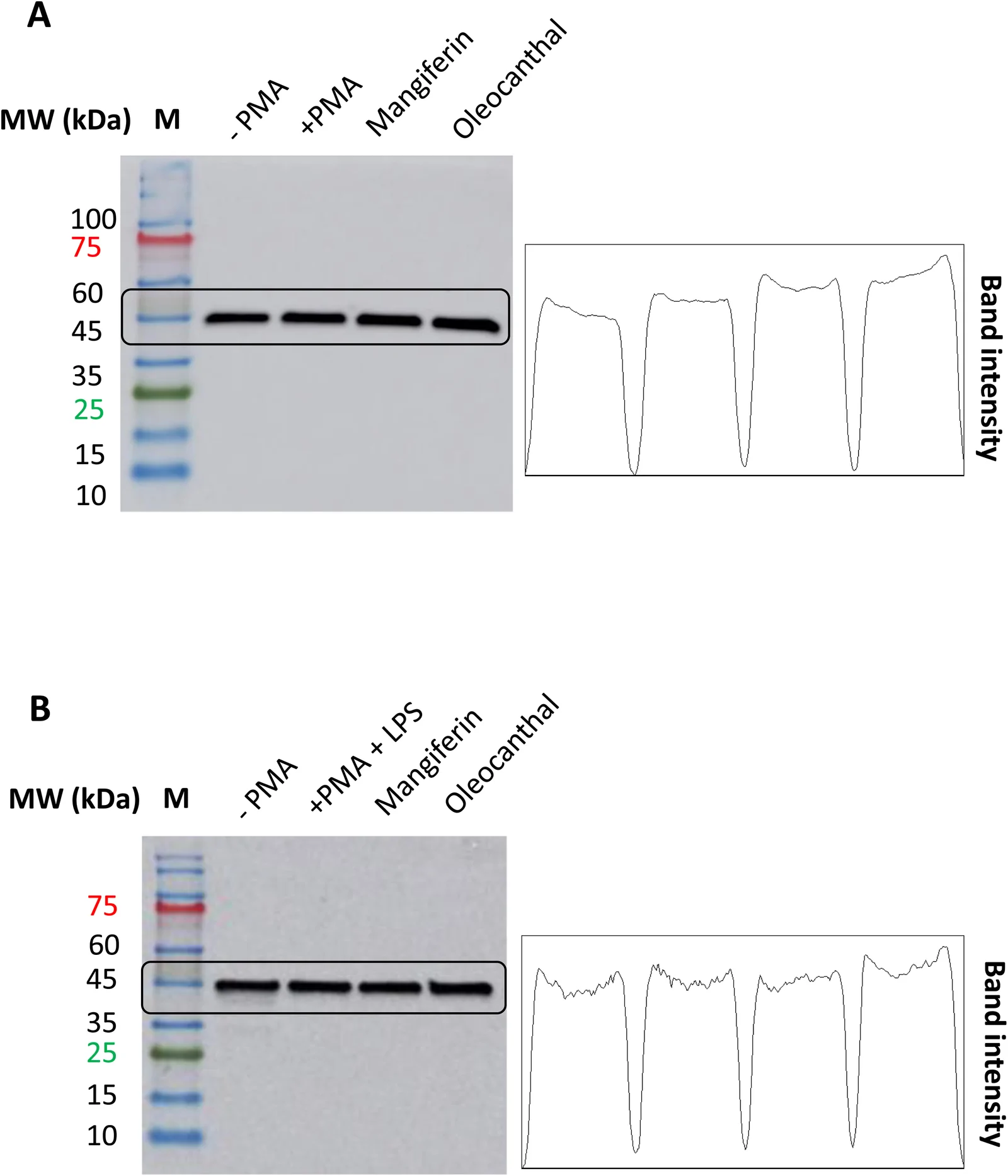
(A) Western blotting performed using an anti-β-actin antibody (molecular weight: 42 kDa) in undifferentiated and differentiated U-937 cells. Lane assignments are as follows: lane 1-molecular weight marker; lane 2-negative control (-PMA); lane 3-positive control (+PMA); lane 4-PMA+ mangiferin; lane 5-PMA+ oleocanthal. In (B) U-937 cells were treated with PMA and LPS, all other conditions remain as in (A).

**Fig. 4:**
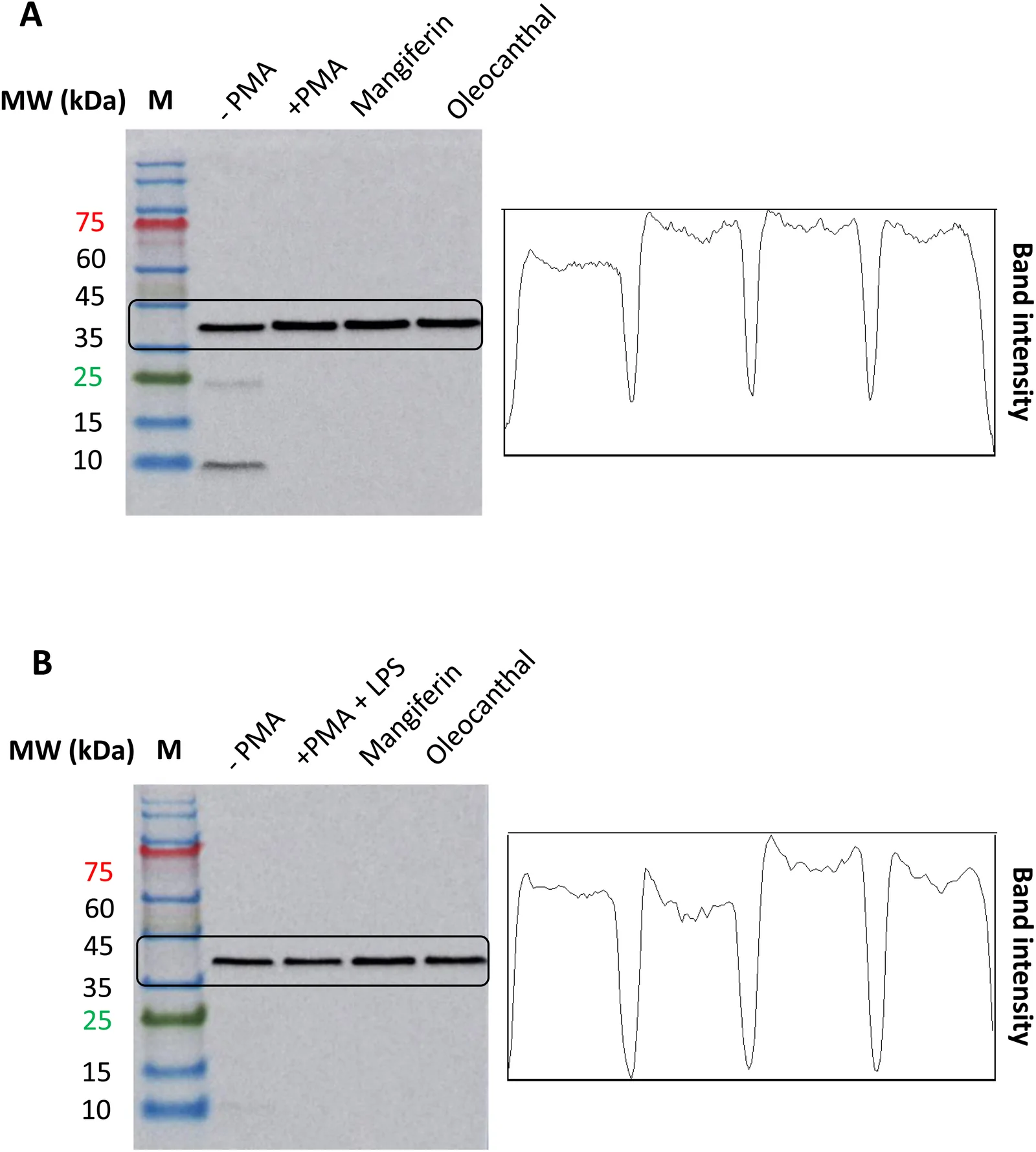
(A) Western blot analysis of GAPDH expression (molecular weight: 36 kDa) in undifferentiated and differentiated U-937 cells. Lane assignments are as follows: lane 1-molecular weight marker; lane 2-negative control (-PMA); lane 3-positive control (+PMA); lane 4-PMA + mangiferin; lane 5-PMA + oleocanthal. In (B) U-937 cells were treated with PMA and LPS, all other conditions remain as in (A).

In protein expression studies, the selection of internal reference genes with stable expression across different experimental conditions is essential. Housekeeping genes, which support basic cellular structure and function, are typically assumed to maintain steady expression under both normal and modified cellular environments. Commonly used reference proteins include β-actin, GAPDH, and tubulin. In the present study, β-actin and GAPDH were selected as reference proteins, and their expression was evaluated in both non-differentiated and differentiated cells across all experimental conditions (Fig. 3 and 4). As part of the experimental design, U-937 cells were treated with different concentrations of PMA for 72 h to induce differentiation, followed by a 24 h incubation with the selected bioactive compounds, mangiferin and oleocanthal. In one experimental set, LPS was used to induce inflammation for 3 h, as described in the Methodology section. Across all conditions in which cells were treated with PMA alone (Fig. 3A) or PMA in combination with LPS (Fig. 3B), β-actin expression differed between non-differentiated and differentiated cells. Specifically, non-differentiated cells exhibited lower β-actin expression, whereas differentiated cells showed higher expression levels, consistent with our previous findings (Fig. 3).^7^ This variation in β-actin expression is likely attributable to cytoskeletal remodeling that occurs during cellular differentiation to meet increased structural and functional demands. GAPDH expression remained relatively stable under all experimental conditions (Fig. 4). The densitogram presented in the right panel illustrates the changes in band intensity.

### 3.4 Effect of bioactive compounds on inflammatory cytokine

**Fig. 5:**
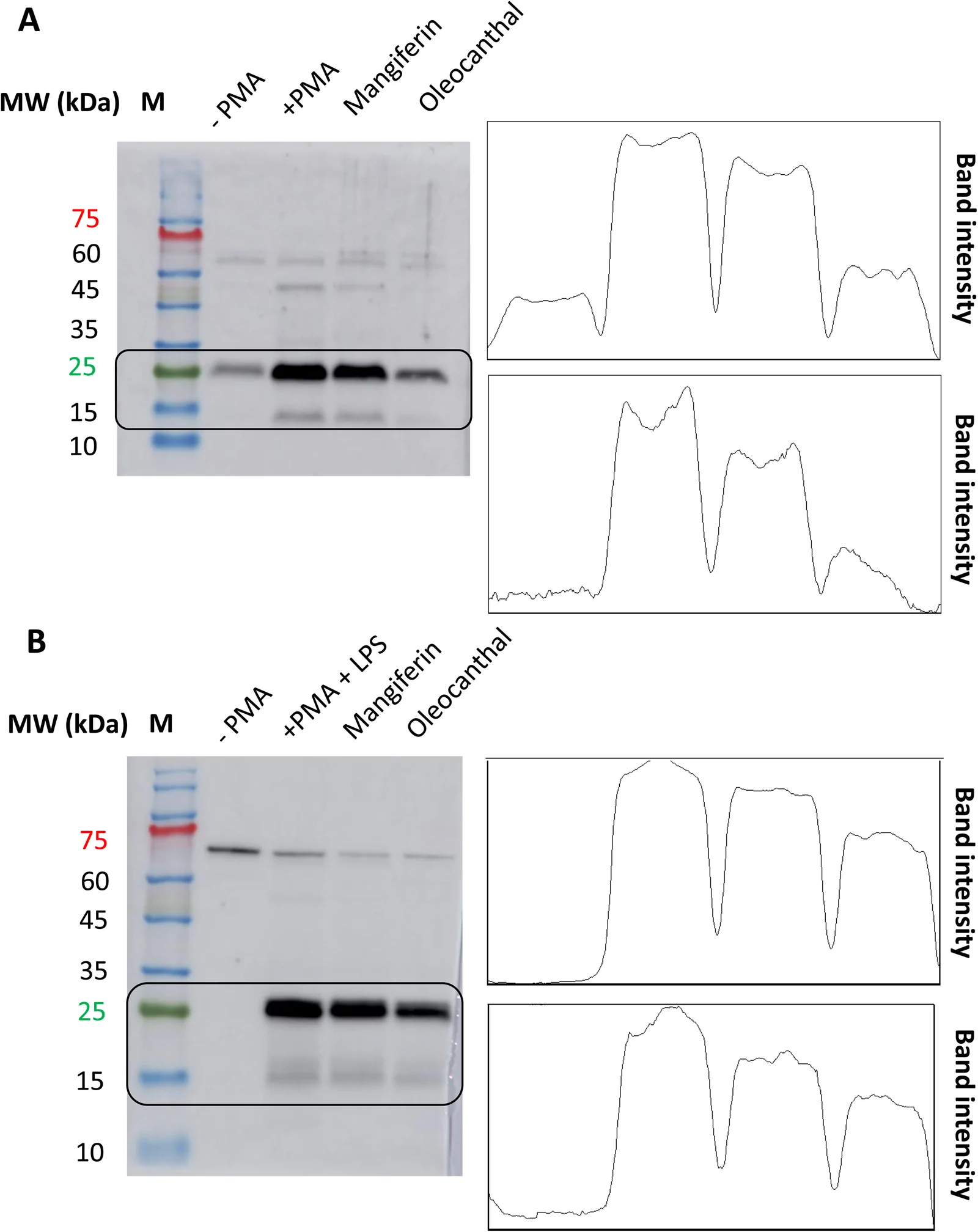
Western blot analysis of TNF-α. (A) Analysis of TNF-α expression. Lane 1: molecular weight marker; lane 2: undifferentiated cells; lane 3: differentiated control cells; lane 4: mangiferin-treated cells; lane 5: oleocanthal-treated cells. (B) U-937 cells were treated with PMA and LPS; all other conditions were the same as in (A).

Upon activation, macrophages are known to produce various pro-inflammatory cytokines, including interleukin-6 (IL-6), TNF-α, and interleukin-6 (IL-12). In this study, we evaluated the effects of selected bioactive compounds on pro-inflammatory responses in cells treated with PMA alone or in combination with LPS (Fig. 5). TNF-α, a key pro-inflammatory cytokine, was markedly downregulated in response to mangiferin and oleocanthal compared to PMA-treated controls. As shown in Fig. 5A, PMA-induced differentiated cells exhibited substantially higher TNF-α levels than non-treated controls. Subsequent treatment with the bioactive compounds resulted in a significant reduction of TNF-α expression, as further illustrated by the densitogram on the right panel. A similar pattern of TNF-α expression was observed in cells treated with PMA and LPS, with the only difference being that TNF-α levels were markedly higher compared to controls (Fig. 5B).

### 3.5 Effect of bioactive compounds on IL-4, an anti-inflammatory cytokine

**Fig. 6:**
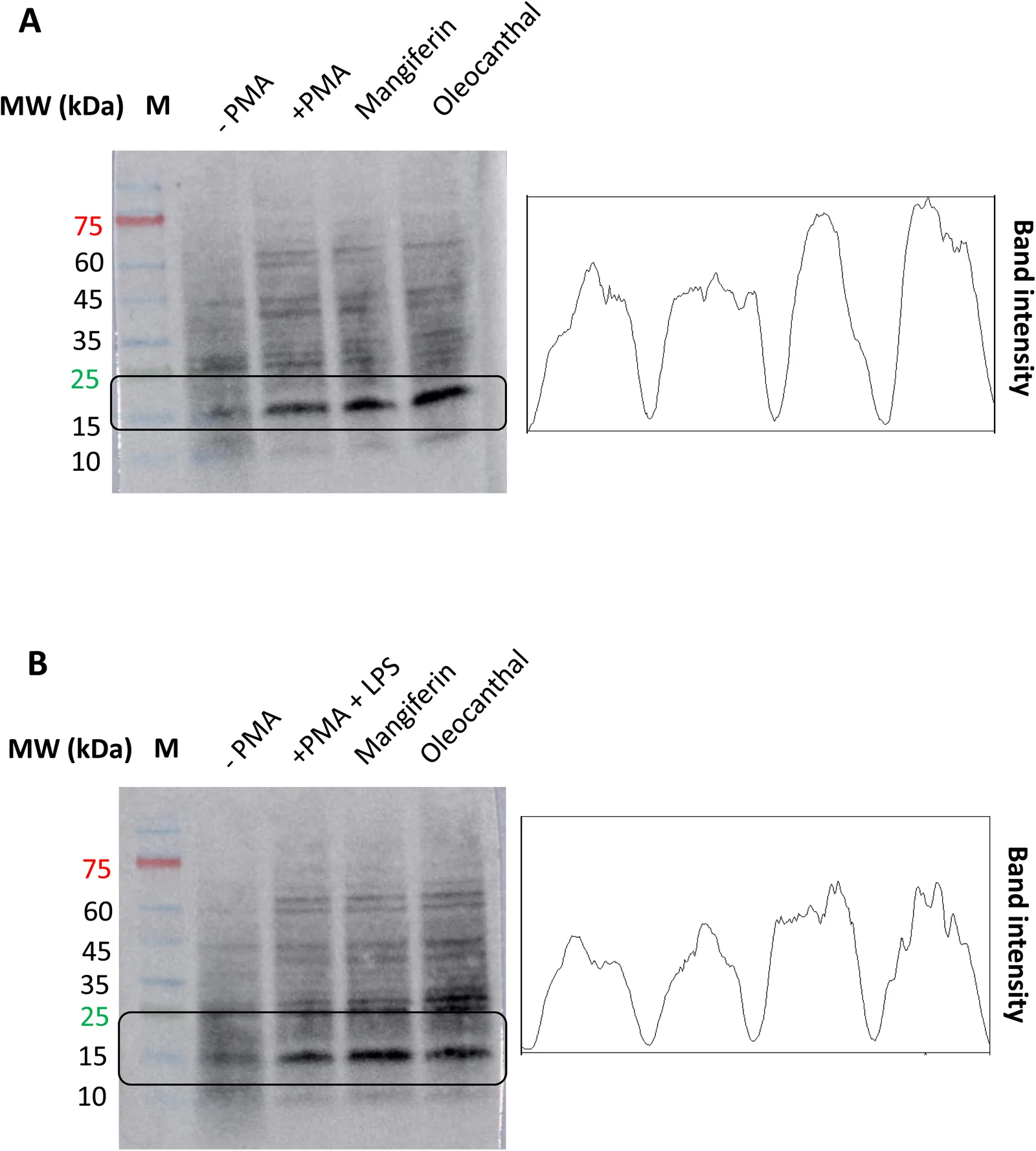
Western blot analysis of IL-4. (A) Analysis of IL-4 expression. Lane 1: molecular weight marker; lane 2: undifferentiated cells; lane 3: differentiated control cells; lane 4: mangiferin-treated cells; lane 5: oleocanthal-treated cells. (B) U-937 cells were treated with PMA and LPS; all other conditions were the same as in (A).

IL-4, an anti-inflammatory cytokine, showed elevated expression in PMA-induced samples treated with mangiferin and oleocanthal compared with the control samples. In addition, IL-4 expressions were markedly increased in LPS-treated samples (Fig. 6). Increased IL-4 expression has been reported to correlate with the suppression of pro-inflammatory cytokines, including TNF-α and IL-12, while promoting the expression of anti-inflammatory cytokines such as IL-10.^34^ These findings suggest that the inhibition of pro-inflammatory cytokine expression is directly associated with the attenuation of the inflammatory response.

### 3.6 Effect of bioactive compounds on 5-LOX expression

**Fig. 7:**
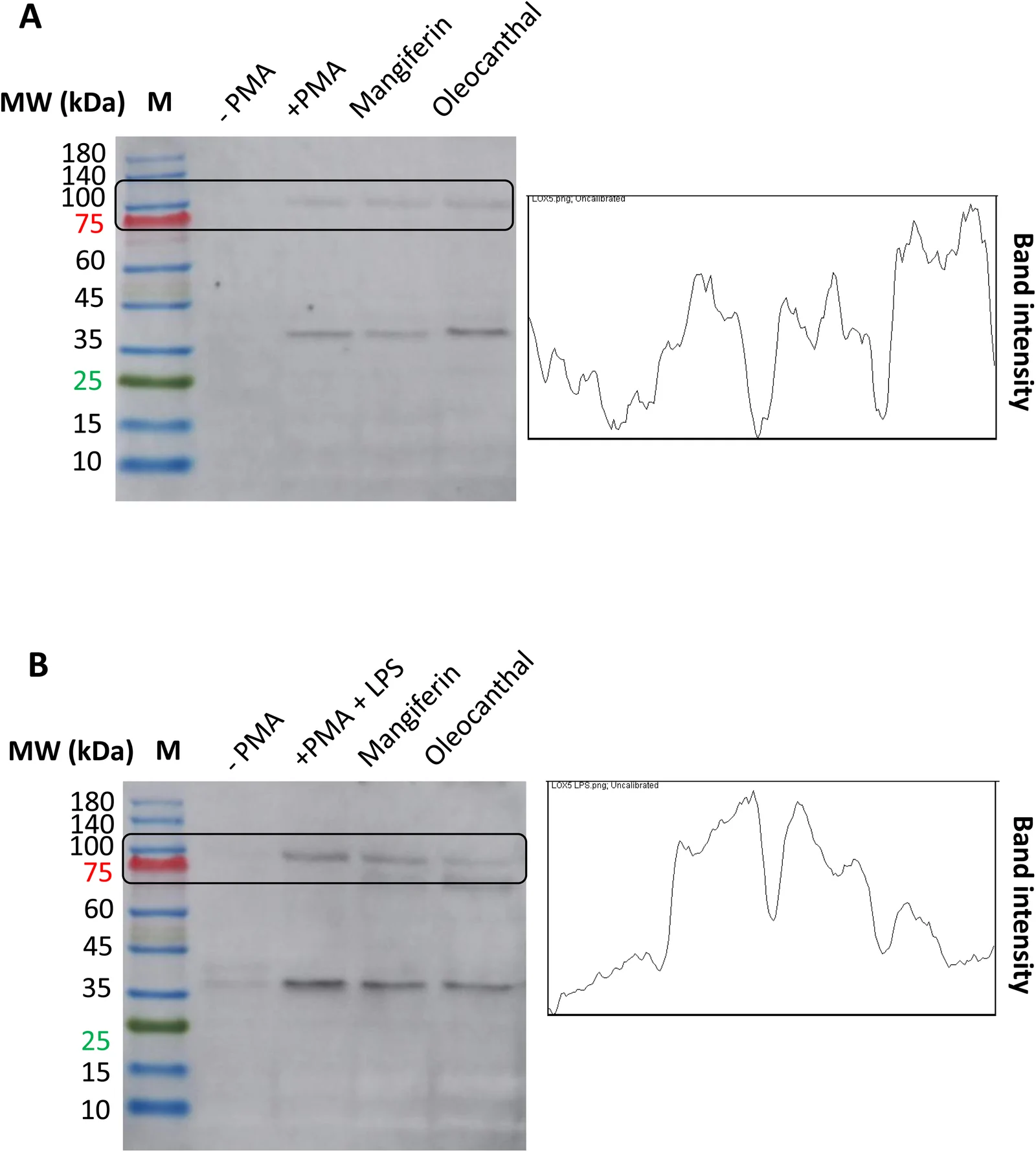
Western blot analysis of 5-LOX. (A) Western blot analysis of 5-LOX expression. Lane 1: molecular weight marker; lane 2: undifferentiated cells; lane 3: differentiated control cells; lane 4: mangiferin-treated cells; lane 5: oleocanthal-treated cells. (B) U-937 cells were treated with PMA and LPS; all other conditions were the same as in (A).

5-Lipoxygenase (5-LOX; ALOX5) is a key enzyme in the arachidonic acid metabolic pathway and plays a central role in the regulation of inflammatory responses.^7^ In our current study, no significant change in 5-LOX expression was observed in PMA-induced U-937 cells following treatment with mangiferin or oleocanthal compared to PMA-treated controls (Fig. 7A), indicating that these bioactive compounds do not substantially affect 5-LOX under baseline differentiation conditions. In contrast, under LPS-induced inflammatory conditions (Fig. 7B), treatment with both mangiferin and oleocanthal led to a reduction in 5-LOX expression, suggesting that these compounds can modulate enzyme levels during acute inflammatory activation. The densitogram presented in the right panel further illustrates these changes in band intensity. Collectively, these findings highlight the potential therapeutic relevance of mangiferin and oleocanthal in regulating 5-LOX mediated inflammatory responses, particularly in scenarios of heightened inflammatory stress induced by LPS. Further studies are warranted to explore the precise molecular mechanisms underlying this inhibitory effect^35^ (Fig. 7).

**Fig. 8:**
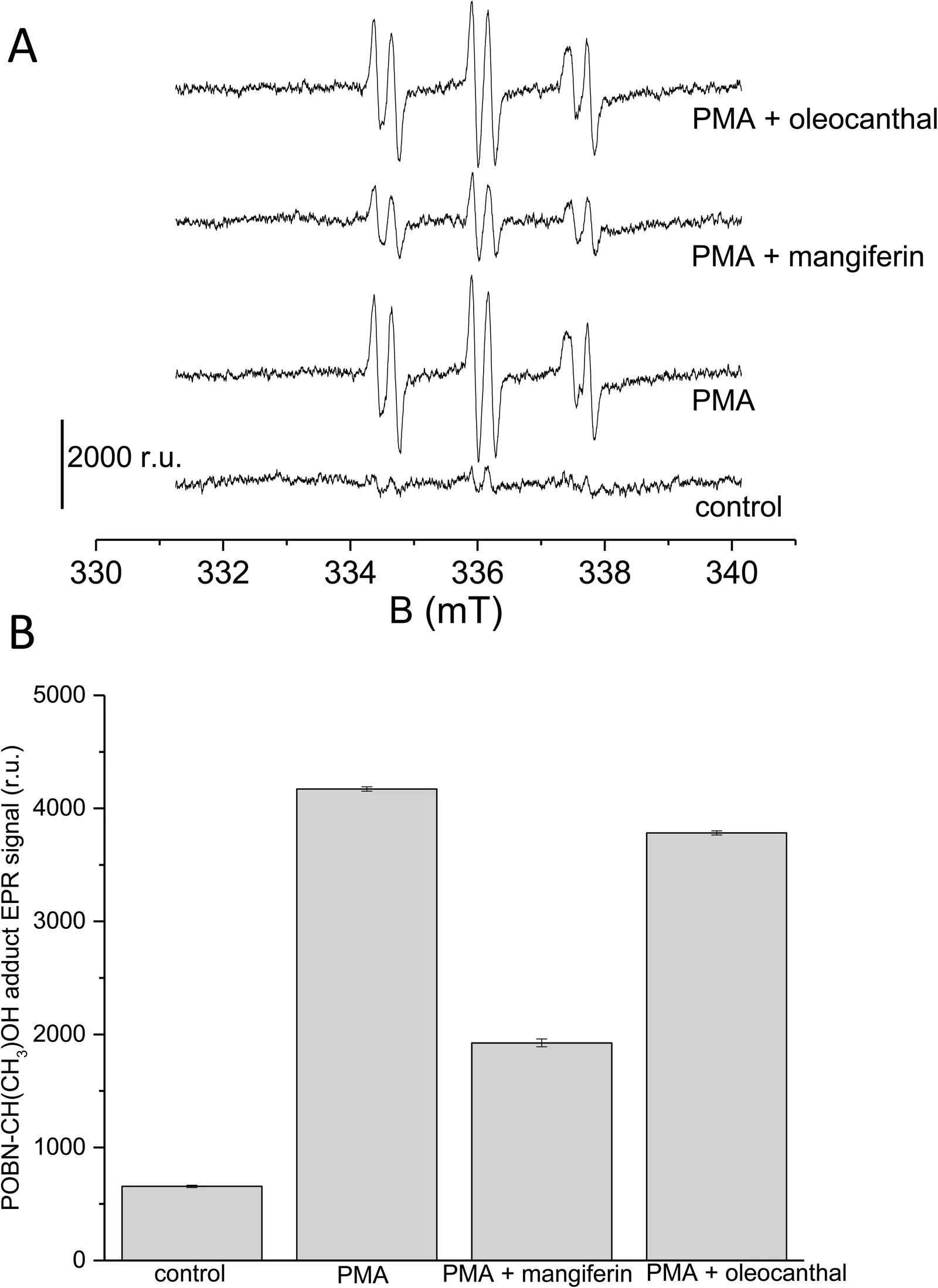
PMA-induced POBN–CH(CH_3_)–OH adduct EPR spectra in U-937 cells. After 72 h of differentiation, U-937 cells were incubated with 25 mM POBN in the absence and presence of bioactive compounds. Mangiferin and oleocanthal (5 µM) were added for 24 h prior to measurement. In (A), the vertical bar represents 2000 relative units, whereas in (B), the relative intensities are presented.

**Fig. 9:**
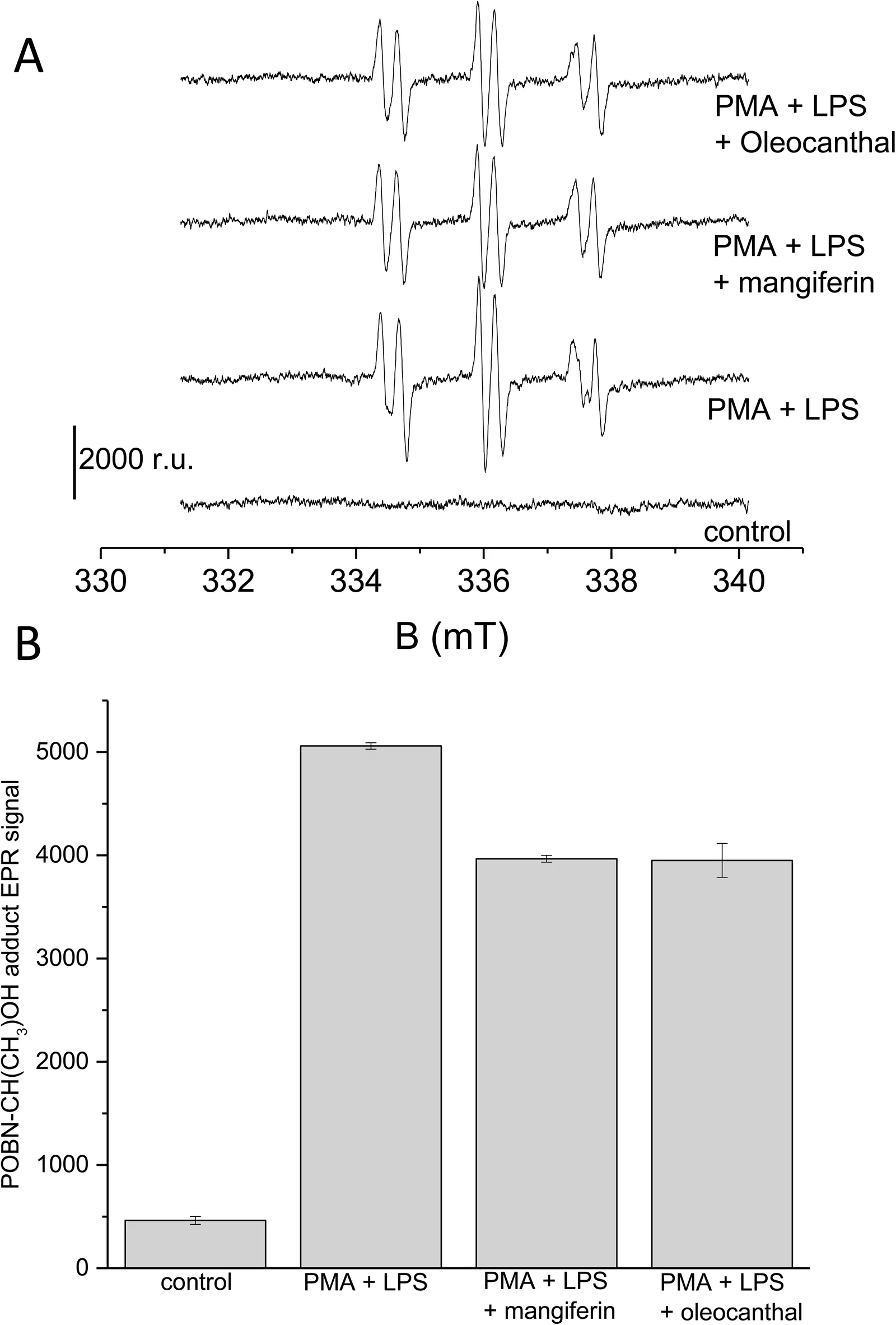
PMA-induced POBN–CH(CH_3_)–OH adduct EPR spectra in U-937 cells. After 72 h of differentiation, U-937 cells were incubated with 25 mM POBN in the presence of 1 µg LPS, in the absence and presence of bioactive compounds. Mangiferin and oleocanthal (5 µM) were added for 24 h, and LPS was added for the last 3 h prior to measurement. In (A), the vertical bar represents 2000 relative units, whereas in (B), the relative intensities are presented.

To validate these findings, EPR measurements were performed to directly assess the suppression of ROS generated by the bioactive compounds. This approach enabled the detection and comparison of ROS-derived radical adducts, allowing evaluation of the capacity of the tested compounds to attenuate ROS formation under differentiation conditions (Fig. 8) and during co-treatment with LPS induction (Fig. 9). No or negligible EPR signal was detected in control U-937 cells, whereas 72 h PMA-induced differentiation resulted in the formation of the α-hydroxyethyl radical adduct of POBN [POBN–CH(CH_3_)–OH], as evidenced by apparent EPR signals following sonication. Treatment with either mangiferin or oleocanthal reduced the EPR signal intensity. A similar trend was observed in LPS-treated U-937 cells, where LPS induction produced a comparatively higher EPR signal, which was again attenuated by both bioactive compounds (Fig. 9).

### 3.7 Impact of bioactive compounds on lipid peroxidation marker (MDA) and protein modification

In the presence of oxidative stress or excessive ROS production, unsaturated lipids undergo a series of oxidative reaction referred to as lipid peroxidation.^34^ A key reactive end product of this process is malondialdehyde (MDA), which can covalently modify proteins to form MDA-protein adducts, thereby serving as a marker of oxidative protein damage/protein modification.^36,37^ In our study, LPS-treated U-937 cells showed elevated levels of MDA, indicating increased lipid peroxidation and oxidative stress, which was further reflected in enhanced MDA-protein adduct formation detected by western blot analysis. In contrast, treatment with oleocanthal reduced MDA accumulation and associated protein adduct formation, whereas mangiferin showed only a slight effect (SI data 3).

## 4. Discussion

In the present study, we investigated the effects of the bioactive compounds mangiferin and oleocanthal on oxidative stress and inflammatory responses using a macrophage-based *in vitro* model. Cell viability, assessed by the trypan blue exclusion assay, demonstrated that treatment with both compounds did not induce any significant cytotoxicity under the experimental conditions (Fig. 1). These findings indicate that mangiferin and oleocanthal are well tolerated at tested concentrations; membrane integrity analysis confirmed the preservation of cellular structure across all treatment groups (Fig. 2 and SI data 2), suggesting that the compounds do not induce apoptosis or cytolysis. Although previous studies have reported dose-dependent cytotoxic effects of these compounds, our results support their cytocompatibility within the selected experimental range.^14^ In addition to their antioxidant and anti-inflammatory properties, both compounds have also been reported to exhibit anticancer potential, further highlighting their therapeutic relevance for future use.^42–44^

**Scheme 1:**
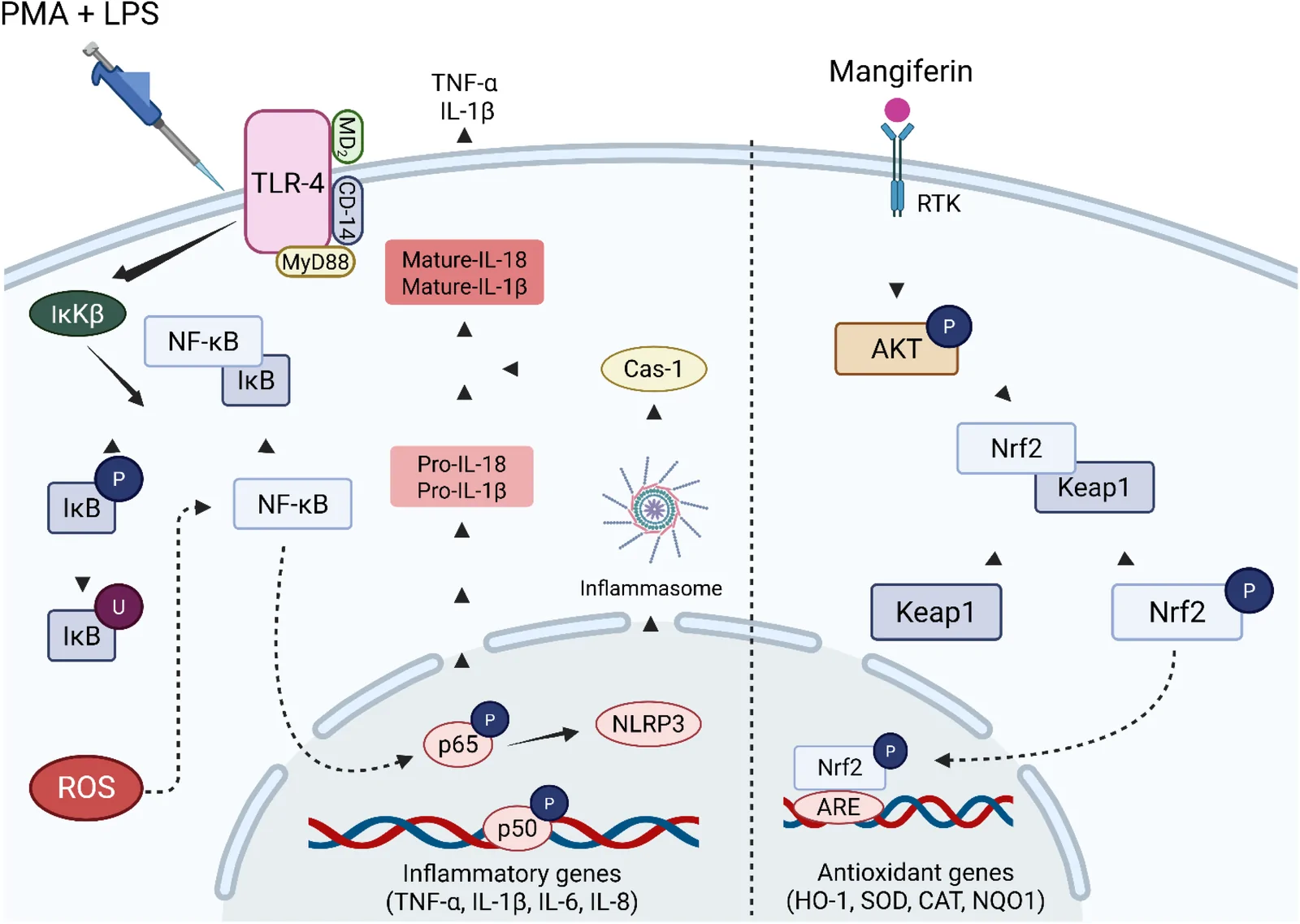
The inflammatory cascade in macrophages induced by PMA and LPS involves activation of the TLR4-MD2-CD14-MyD88 complex, which stimulates IκK, leading to dissociation of IκB from NF-κB. NF-κB then translocates to the nucleus to drive transcription of inflammatory genes, including TNF-α, IL-1β, IL-6, and IL-8. It also upregulates NLRP3, promoting inflammasome assembly and recruitment of caspase-1, which cleaves pro-IL-1β and pro-IL-18 into their mature forms. The released IL-1β and IL-18 sustain a proinflammatory microenvironment and reinforce inflammation in neighboring cells. In parallel, LPS-induced ROS further activate NF-κB, amplifying this inflammatory loop, while secreted IL-1β can further stimulate IκK *via* TLRs, strengthening a feed-forward cycle. Mangiferin suppresses this cascade by activating AKT phosphorylation *via* receptor tyrosine kinases, promoting dissociation of Keap1 from Nrf2. This enables Nrf2 nuclear translocation and induction of antioxidant genes (HO-1, SOD, CAT, NQO1). The resulting antioxidant enzymes reduce ROS accumulation, thereby inhibiting both NF-κB activation and NLRP3 inflammasome activity. Consequently, mangiferin attenuates production of TNF-α, IL-1β, IL-6, and IL-8, and shifts macrophages toward an antioxidant, inflammasome-suppressing phenotype (prepared using https://biorender.com).

**Scheme 2:**
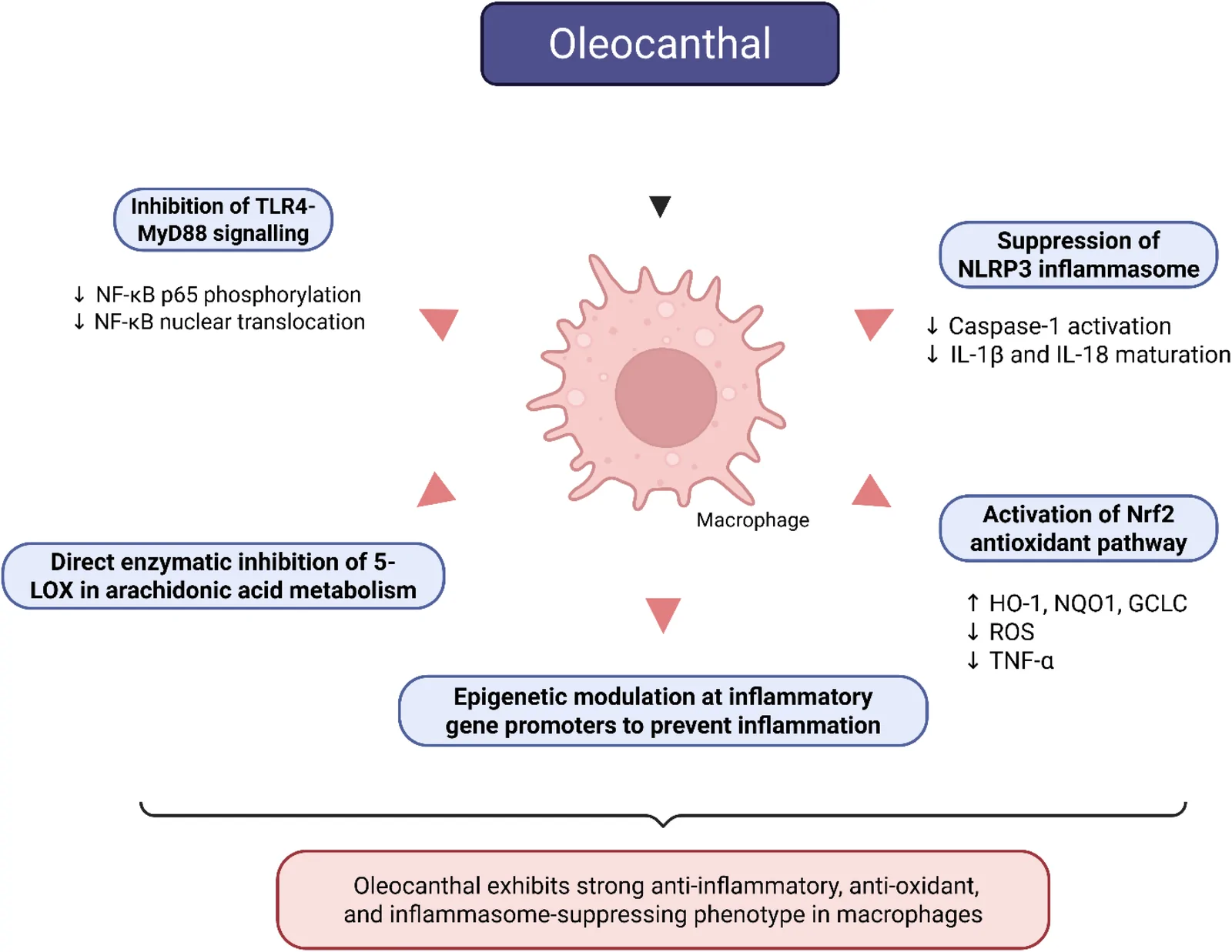
The molecular mechanisms of oleocanthal and its anti-inflammatory and antioxidant effects in macrophages involve inhibition of TLR4-MyD88 signaling, leading to reduced NF-κB-p65 phosphorylation and blockade of nuclear translocation, thereby suppressing transcription of proinflammatory cytokines. It also downregulates NLRP3 inflammasome activity by decreasing caspase-1 activation and maturation of IL-1β and IL-18. In parallel, oleocanthal activates the Nrf2 pathway, increasing expression of antioxidant genes (HO-1, NQO1, GCLC) and reducing ROS and TNF-α levels. Additionally, it directly suppresses 5-LOX in arachidonic acid metabolism and contributes to epigenetic modulation at inflammatory gene promoters. Collectively, these effects promote an anti-inflammatory, antioxidant, and inflammasome-suppressive macrophage phenotype (prepared using https://biorender.com).

Macrophages and their precursor monocytes are central mediators of inflammatory responses and play key roles in innate immunity and the pathogenesis of chronic diseases, including diabetes and Alzheimer's disease. Upon activation, macrophages produce pro-inflammatory and anti-inflammatory cytokines such as TNF-α and IL-4, as well as lipid-metabolizing enzymes including 5-lipoxygenase (5-LOX), which catalyzes leukotriene biosynthesis and amplifies inflammatory signaling cascades (Scheme 1 and 2).^45^ Dysregulation of these pathways contributes to sustained inflammation and disease progression, making macrophages critical targets for anti-inflammatory strategies.^45^ To mimic inflammation under controlled conditions, we utilized the human monocytic U-937 cell line, a well-established system for studying macrophage-like responses due to its reproducible differentiation and robust responsiveness to LPS stimulation. In our study, LPS stimulation resulted in increased inflammatory signaling, as evidenced by elevated TNF-α expression (Fig. 5) and activation of 5-LOX-associated pathways (Fig. 7), consistent with previously reported observations.^45^ These results confirm the suitability of U-937 cells as a model for evaluating inflammation-driven molecular mechanisms and screening anti-inflammatory compounds.^46^ TNF-α is a key regulator of inflammatory signaling, exerting its effects through TNFR1-and TNFR2-mediated pathways. It promotes activation of T and B lymphocytes and modulates macrophages and natural killer (NK) cells *via* chemotactic signaling, thereby linking innate and adaptive immune responses (Scheme 1). Furthermore, TNF-α induces prostaglandin synthesis, acute-phase protein production, and cytokine and chemokine expression, while also activating endothelial cells to facilitate vascular responses during inflammation.^8^ At the cellular level, TNF-α activates NF-κB and MAPK signaling pathways in monocytes and macrophages, leading to increased secretion of pro-inflammatory mediators such as IL-6 and IL-1β. In endothelial cells, it enhances the expression of adhesion molecules including ICAM-1 and VCAM-1, promoting leukocyte recruitment and vascular inflammation.^47^ Its effects are highly context-dependent, influencing processes such as apoptosis, cytokine release, and cellular differentiation across different cell types.^48,49^ In this context, our experimental findings demonstrate that treatment with mangiferin and oleocanthal significantly modulates TNF-α expression, indicating a pronounced anti-inflammatory effect. This observation is consistent with previous reports showing that mangiferin suppresses genes associated with inflammation and oxidative stress, thereby attenuating TNF-α-mediated responses. Similarly, oleocanthal exhibits anti-inflammatory activity comparable to non-steroidal anti-inflammatory drugs and has been shown to modulate inflammatory enzyme pathways, including 5-LOX.^23^

The anti-inflammatory activity observed in our study was also associated with modulation of IL-4 expression (Fig. 6). IL-4 is a major anti-inflammatory cytokine involved in macrophage polarization toward the M2 phenotype, which contributes to tissue repair, suppression of inflammatory cytokine production, and resolution of inflammation. Treatment with mangiferin and oleocanthal enhanced IL-4-associated responses, suggesting that these bioactive compounds not only suppress pro-inflammatory pathways but also promote anti-inflammatory signaling mechanisms. Previous studies have similarly demonstrated that natural polyphenolic compounds can increase IL-4 expression while reducing inflammatory mediators and oxidative stress markers.^38,39^ Therefore, the observed increase in IL-4 activity further supports the immunomodulatory and anti-inflammatory potential of mangiferin and oleocanthal.

The 5-LOX pathway represents another critical component of the inflammatory response. This enzyme catalyzes the conversion of arachidonic acid into leukotriene precursors, which are further metabolized into bioactive lipid mediators that regulate immune cell recruitment, vascular permeability, and cytokine production. In our study, modulation of 5-LOX activity was observed following treatment with the tested compounds (Fig. 7), supporting their role in regulating lipid-mediated inflammatory signaling. Given that 5-LOX is predominantly expressed in monocytes and macrophages, its involvement further validates the use of U-937 cells for investigating inflammation-associated pathways.^45^ Dysregulation of 5-LOX has been implicated in multiple inflammatory diseases, including asthma, arthritis, cardiovascular disorders, and cancer, emphasizing its relevance as a therapeutic target. Additionally, mangiferin and oleocanthal significantly reduced MDA levels compared with the LPS-treated control group, indicating attenuation of lipid peroxidation and oxidative damage (SI data 3). Malondialdehyde is one of the final products of polyunsaturated fatty acid oxidation and serves as an important biomarker for oxidative stress-related cellular injury. The reduction in MDA observed in our study suggests that both compounds possess substantial antioxidant activity capable of limiting ROS-mediated membrane damage. These findings agree with previous studies reporting that mangiferin and oleocanthal exert potent free radical scavenging effects and protect cells against oxidative stress-induced injury.^40,41^

Importantly, the combined assessment of TNF-α expression (Fig. 5), IL-4 expression (Fig. 6) and 5-LOX activity (Fig. 7), and oxidative stress parameters provide an integrated view of the inflammatory response in this model system. Our data demonstrates that mangiferin and oleocanthal act as effective modulators of macrophage-mediated inflammation by targeting both cytokine signaling and lipid inflammatory pathways.

These findings are supported by previous pharmacological studies targeting macrophage-driven inflammation. For example, berberine has been shown to suppress TNF-α production, ROS generation, and inflammatory gene expression in macrophage models without inducing cytotoxicity.^50^ Similarly, the CDK8/19 inhibitor Senexin B reduced cytokine production and inflammatory responses in monocyte-derived cell lines.^51^ In neuroinflammatory contexts, modulation of macrophage-related pathways, such as TREM2 signaling, has been shown to regulate cytokine release and enhance phagocytic activity.^52^ Collectively, these studies reinforce the central role of macrophages in inflammation and highlight multiple therapeutic strategies for modulating their function. Using this model our study demonstrates that mangiferin and oleocanthal as a potential modulator of macrophage mediated inflammation, offering insights into their therapeutic potential for controlling inflammatory responses.

## 5 Conclusion

This study provides valuable insights into the anti-inflammatory potential of plant-derived bioactive compounds and highlights promising avenues for future research. Mangiferin and oleocanthal demonstrated significant anti-inflammatory and antioxidant activities in LPS-stimulated differentiated U-937 cells. Both compounds modulated inflammatory responses through suppression of pro-inflammatory mediators and enhancement of anti-inflammatory signaling. In addition, treatment with mangiferin and oleocanthal significantly reduced oxidative stress markers such as MDA, indicating attenuation of lipid peroxidation and cellular oxidative damage. These findings were supported by EPR analysis, which confirmed the direct suppression of ROS-derived radical formation by both bioactive compounds under PMA and PMA/LPS-induced conditions. Collectively, the results suggest that mangiferin and oleocanthal exert protective effects through combined antioxidant and immunomodulatory mechanisms, highlighting their potential as promising natural therapeutic agents for inflammation-associated oxidative stress disorders.

## Conflicts of interest

The authors declare that they have no competing interests.

## Supplementary Material

RA-016-D6RA01563H-s001

RA-016-D6RA01563H-s002

RA-016-D6RA01563H-s003

RA-016-D6RA01563H-s004

## Data Availability

All data generated or analysed during this study are included in this published article and its supplementary information (SI). Supplementary information is available. See DOI: https://doi.org/10.1039/d6ra01563h.
