# Supplementary material for: Mangiferin and oleocanthal in the modulation of oxidative stress in monocytes and macrophages
Source: RSC Adv. 2026 Jul 8;16(40):45788–803. doi: 10.1039/d6ra01563h (PMC13343847; doi:10.1039/d6ra01563h)
Supplement: Supplementary file 1 [file RA-016-D6RA01563H-s001.pdf]

This journal is © The Royal Society of Chemistry 2026

Supplementary Data 1: Detailed information of the primary and secondary antibody used for the western blotting analysis.

Supplementary Data 2: Evaluation of cell viability and membrane integrity using dual staining with SYTO9 and PI. (A) Non-treated U-937 cells and cells treated with PMA or PMA + bioactive compounds were stained, and (B) non-treated U-937 cells and cells treated with PMA + LPS or bioactive compounds (together with PMA and LPS) were stained with SYTO9 and PI and analysed using confocal microscopy. SYTO9-positive cells exhibited green fluorescence, whereas PI-positive cells exhibited yellow/orange fluorescence.

Supplementary Data 3: Western blot analysis of Protein malondialdehyde (Protein-MDA) adduct **A.** Lane 1: molecular weight marker; Lane 2: undifferentiated cells; Lane 3: differentiated control cells; Lane 4: mangiferin-treated cells; Lane 5: oleocanthal-treated cells. **B.** U-937 cells were treated with PMA and LPS; all other conditions were the same as in (A).
