# Supplementary material for: Mangiferin and oleocanthal in the modulation of oxidative stress in monocytes and macrophages
Source: RSC Adv. 2026 Jul 8;16(40):45788–803. doi: 10.1039/d6ra01563h (PMC13343847; doi:10.1039/d6ra01563h)
Supplement: Supplementary file 4 [file RA-016-D6RA01563H-s004.pdf]

Supplementary Information (SI) for RSC Advances.
This journal is © The Royal Society of Chemistry 2026

Supplementary data 3

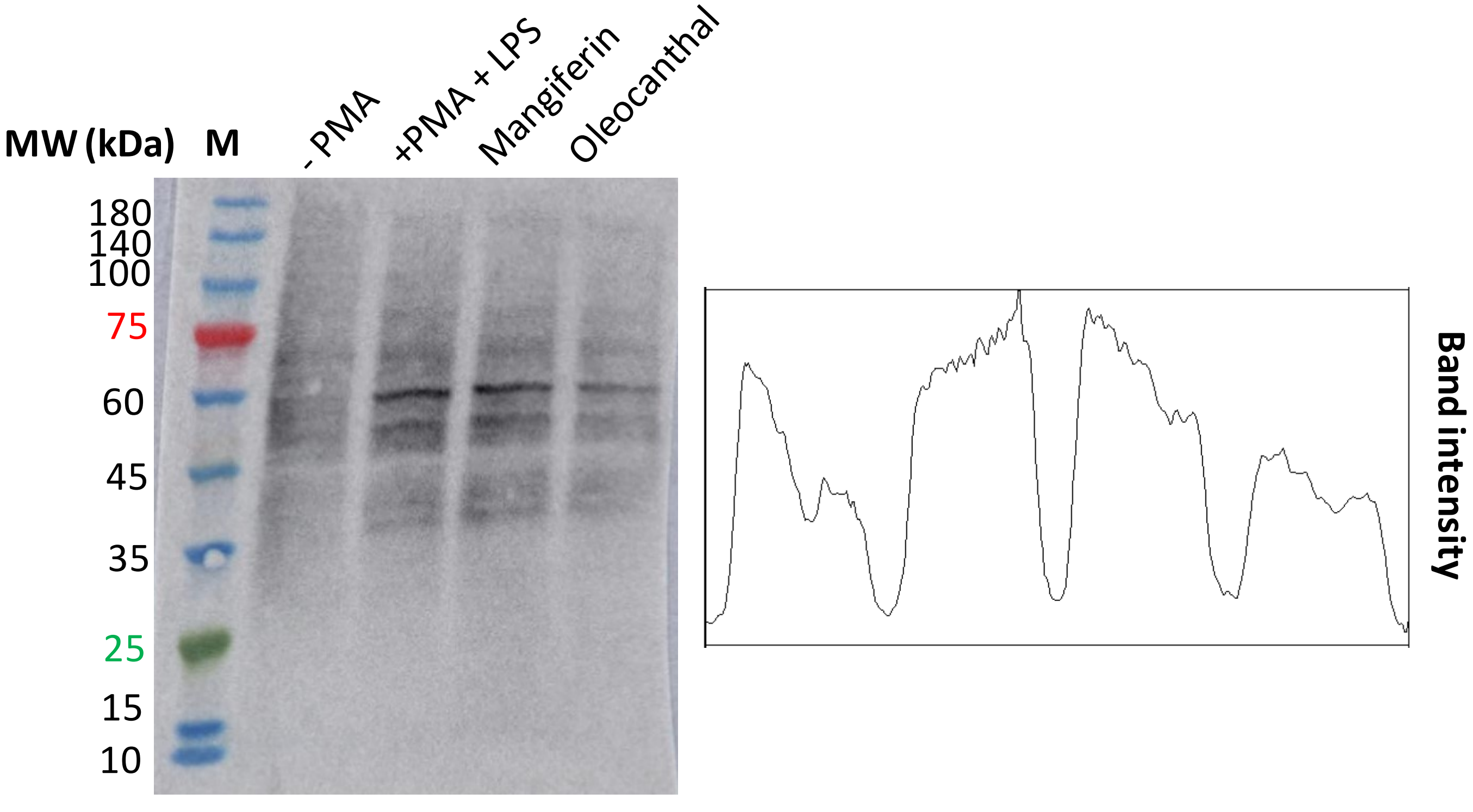

Supplementary Data 3: Western blot analysis of Protein malondialdehyde (Protein-MDA) adduct **A.** Lane 1: molecular weight marker; Lane 2: undifferentiated cells; Lane 3: differentiated control cells; Lane 4: mangiferin-treated cells; Lane 5: oleocanthal-treated cells. **B.** U-937 cells were treated with PMA and LPS; all other conditions were the same as in (A).
