# Supplementary material for: Mangiferin and oleocanthal in the modulation of oxidative stress in monocytes and macrophages
Source: RSC Adv. 2026 Jul 8. Online ahead of print. doi: 10.1039/d6ra01563h (PMC13343847; doi:10.1039/d6ra01563h)
Supplement: RA-OLF-D6RA01563H-s002 [file RA-OLF-D6RA01563H-s002.pdf]

Supplementary Information (SI) for RSC Advances.
This journal is © The Royal Society of Chemistry 2026

Supplementary data 1

Name	Catalogue no.	Host animal	MW	Antibody dilution
**IL-4**	ab9622	Rabbit polyclonal	17 kDa	1:2500
**LOX-5**	66326-1-Ig	Mouse monoclonal	70-78 kDa	1:4000
**TNF-α**	60291-1-Ig	Mouse monoclonal	26 kDa	1:4000
**MDA**	ab27642	Rabbit polyclonal	-----	1:5000
**β-actin**	66009-1-Ig	Mouse monoclonal	42 kDa	1:25000
**GAPDH**	60004-1-Ig	Mouse monoclonal	36 kDa	1:20000
**HRP-conjugated**	SA00001-1	Goat anti-mouse	-----	1:10000
**HRP-conjugated**	SA00001-2	Goat anti-rabbit	-----	1:10000

Supplementary Data 1: Detailed information of the primary and secondary antibody used for the western blotting analysis.
