# Supplementary material for: Mangiferin and oleocanthal in the modulation of oxidative stress in monocytes and macrophages
Source: RSC Adv. 2026 Jul 8. Online ahead of print. doi: 10.1039/d6ra01563h (PMC13343847; doi:10.1039/d6ra01563h)
Supplement: RA-OLF-D6RA01563H-s003 [file RA-OLF-D6RA01563H-s003.pdf]

Supplementary Information (SI) for RSC Advances.
This journal is © The Royal Society of Chemistry 2026

Supplementary data 2

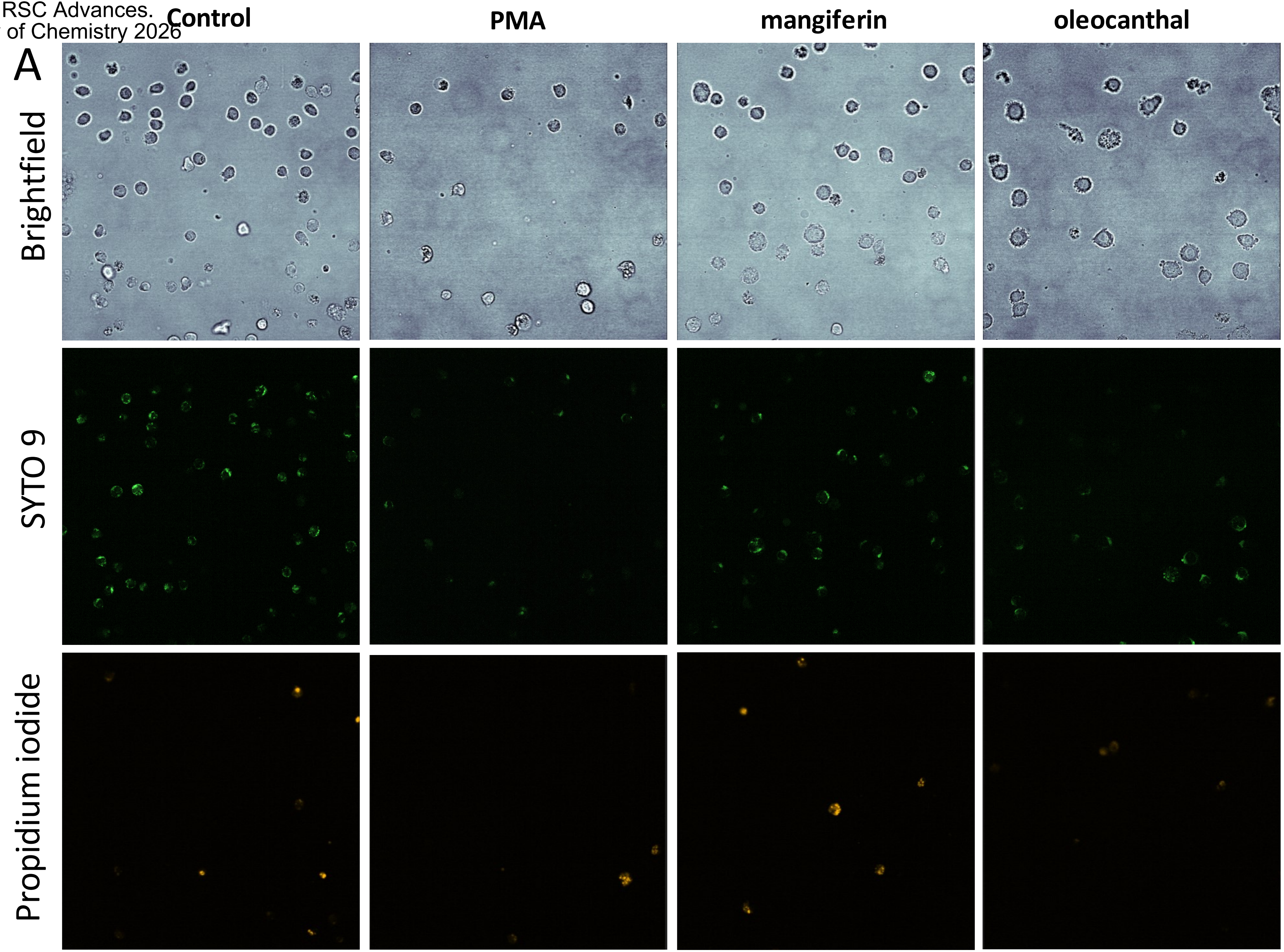

Supplementary data 2

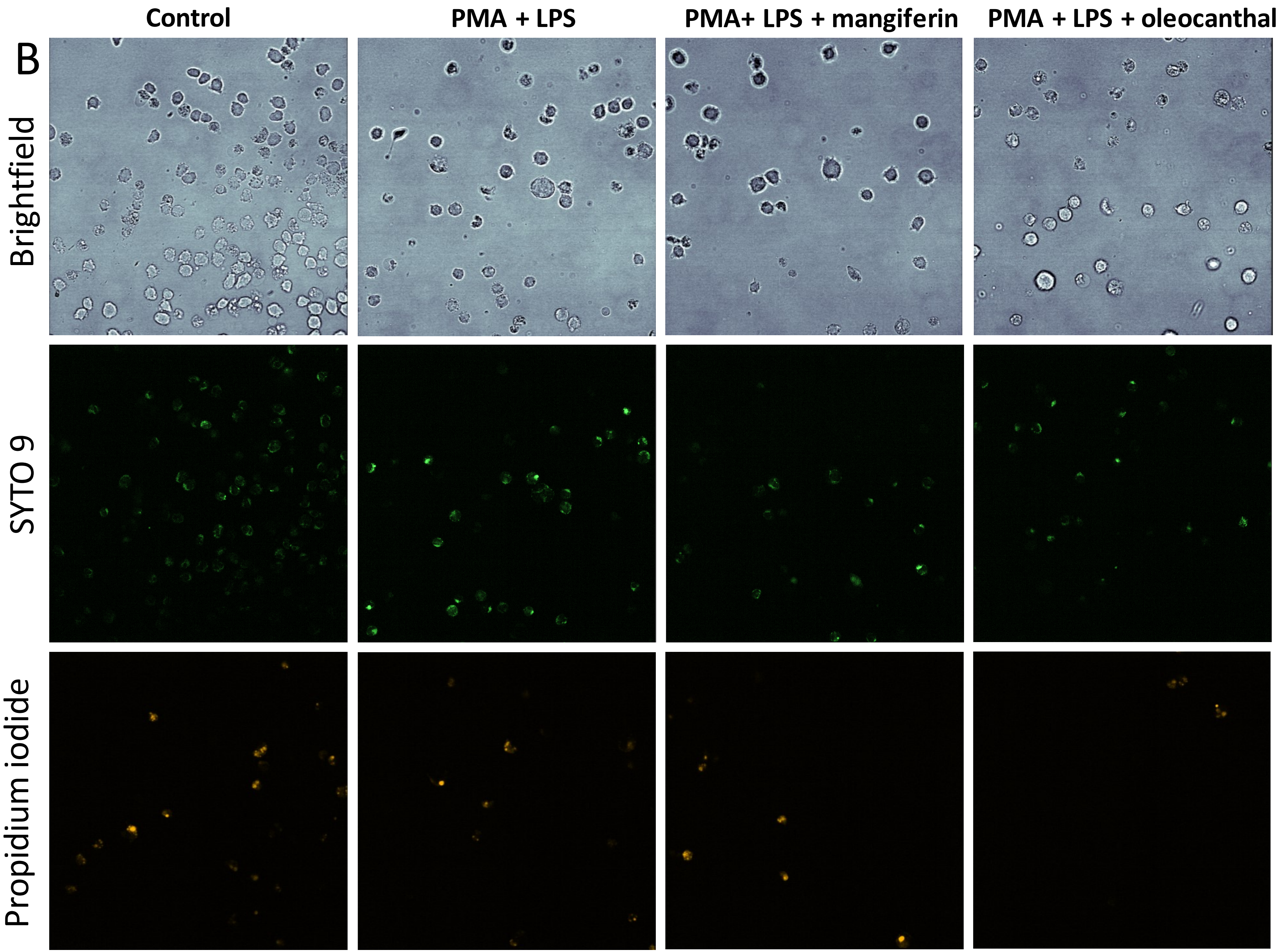

Supplementary Data 2: Evaluation of cell viability and membrane integrity using dual staining with SYTO9 and PI. (A) Non-treated U-937 cells and cells treated with PMA or PMA + bioactive compounds were stained, and (B) non-treated U-937 cells and cells treated with PMA + LPS or bioactive compounds (together with PMA and LPS) were stained with SYTO9 and PI and analysed using confocal microscopy. SYTO9-positive cells exhibited green fluorescence, whereas PI-positive cells exhibited yellow/orange fluorescence.
